# Delirium-Tremens

**Published:** 1874-05

**Authors:** N. S. Davis


DELIRIUM TREMENS.—*By N. S. Davis, M.D.*—The patient should be kept at rest, with kind, persuasive, encouraging words, and as little physical or forcible restraint as possible. All alcoholic drinks should be entirely discarded, and, in their place, such medicines given as will exert a soothing, tranquilizing influence, favoring sleep at night; and such bland nourishment as will be most readily retained and assimilated. From ten to fifteen grs. bromide of potassium, given in solution with the same number of minims of the tincture of digitalis, every two or three hours, according to the degree of excitement, and from fifteen to twenty grains of hydrate of chloral, between eight and nine o'clock in the evening, will be all the medicine needed in most of these cases. Nourishment is of even more importance than medicine. We cannot but regard twenty, thirty or forty grain doses of chloral, half grain, and grain doses of morphine, or fluid drachm doses of tincture of digitalis, as dangerous and unnecessary. I have never resorted to such doses; but several cases have come under my observation in which they have been resorted to, some of which terminated suddenly fatal.

